# GnRH antagonist protocol versus long-acting GnRH agonist protocol on pregnancy outcomes in IVF/ICSI: a retrospective cohort study

**DOI:** 10.1080/07853890.2025.2564279

**Published:** 2025-09-30

**Authors:** Panpan Zhao, Xiliang Wang, Qian Zhang, Yinling Xiu, Kaixuan Sun, Kaiyue Wang, Li Yan, Yuexin Yu

**Affiliations:** ^a^ Department of Reproductive Medicine, General Hospital of Northern Theater Command, Shenyang, China; ^b^ Postgrad Training Base General Hospital Northern Theater, Jinzhou Medical University, Shenyang, China

**Keywords:** GnRH antagonist, long-acting GnRH agonist, in-vitro fertilization, assisted reproductive technology, infertility

## Abstract

**Background:**

Gonadotropin-releasing hormone (GnRH) antagonist and long-acting GnRH agonist regimens are commonly employed for ovarian stimulation in women undergoing *in-vitro* fertilization (IVF)/intracytoplasmic sperm injection (ICSI), but their relative efficacy in improving pregnancy outcomes remains controversial. Previous studies has yielded conflicting results, leaving clinicians and researchers uncertain about which protocol is more favorable. Given the high prevalence of infertility and the importance of improving pregnancy outcomes for these patients, understanding the differences in the efficacy of these two protocols is crucial. Therefore, our study aims to conduct a direct comparison of pregnancy outcomes between patients using GnRH antagonist protocol or long-acting GnRH agonist protocol.

**Methods:**

This retrospective study included 575 women with primary infertility who underwent IVF/ICSI at our hospital between January 2021 and December 2022. The study included 501 women who underwent the GnRH antagonist protocol and 74 who underwent the long-acting GnRH agonist protocol. The investigated outcomes included biochemical pregnancy, clinical pregnancy, abnormal pregnancy, miscarriage, and live birth rates.

**Results:**

Univariate analysis found no significant differences between the GnRH antagonist and long-acting GnRH agonist protocols for biochemical pregnancy rate, clinical pregnancy rate, abnormal pregnancy rate, and miscarriage rate, whereas the GnRH antagonist protocol was associated with a lower live birth rate than the long-acting GnRH agonist protocol. After adjusting for potential confounding factors, no significant differences were found between the two protocols for biochemical pregnancy, clinical pregnancy, abnormal pregnancy, miscarriage, and live birth rates.

**Conclusion:**

Our study results show that the GnRH antagonist and long-acting GnRH agonist protocols yielded comparable outcomes in terms of pregnancy outcomes. Further large-scale prospective studies are required to confirm these findings.

## Introduction

Infertility is characterized by the absence of clinical pregnancy after 1 year of regular unprotected sexual intercourse, and it affects 8–12% of couples of childbearing age worldwide [1]. Assisted reproductive technology (ART), including *in-vitro* fertilization (IVF) and intracytoplasmic sperm injection (ICSI), was developed and has solved unwanted non-conception problems in over 60% of young couples [2,3]. Controlled ovarian stimulation protocols are important in improving pregnancy rates for women with infertility undergoing IVF/ICSI [4,5]. The most common controlled ovarian stimulation protocols are the gonadotropin-releasing hormone (GnRH) antagonist and long-acting GnRH agonist protocols [6].

The GnRH antagonist protocol is widely used in clinical practice because it can shorten the stimulation duration, reduce the incidence of ovarian hyperstimulation syndrome, and improve adenomyosis and endometriosis [7–9]. Nowadays, the long-acting GnRH agonist protocol is considered the mainstream controlled ovarian stimulation protocol in China because it can improve endometrial receptivity and increase clinical pregnancy rates in fresh IVF cycles [10–13]. Both protocols have certain advantages; however, prior studies have reported inconsistent results on live birth rates between the GnRH antagonist and long-acting GnRH agonist protocols [14–16]. The optimal treatment protocol for women with infertility undergoing IVF/ICSI remains unclear. Therefore, this study aimed to compare pregnancy outcomes between the GnRH antagonist and long-acting GnRH agonist protocols in women with primary infertility undergoing IVF/ICSI.

## Materials

### Study design and population

This retrospective cohort study included women with primary infertility who underwent their first IVF/ICSI cycle between January 2021 and December 2022 at our hospital. Women with primary infertility who received controlled ovarian stimulation protocols with either the GnRH antagonist or long-acting GnRH agonist protocol were eligible for our study, and all patients in this study underwent frozen embryo transfer. Exclusion criteria included [1]: uterine malformation, intrauterine lesions, hydrosalpinx, or polycystic ovary syndrome [2], uncontrolled systemic diseases [3], endometriosis, adenomyosis, or pelvic inflammatory disease [4], abnormal parental karyotyping [5], secondary infertility, or [6] incomplete data regarding controlled ovarian stimulation protocols or pregnancy outcomes. Controlled ovarian stimulation protocols were selected for women with primary infertility based on age, body mass index (BMI), antral follicle count, and menstrual cycle status. Written informed consent was obtained from all the patients, and the study protocol was reviewed and approved by the Ethics Committee of the General Hospital of the Northern Theater Command.

### Long-acting GnRH agonist protocol

For women who underwent the GnRH-agonist protocol, a long-acting GnRH-agonist (3.75 mg) was subcutaneously injected for ≥ 1 time on 2–5 days of the menstrual cycle. Specifically, this means that a single dose of 3.75 mg of the long-acting GnRH-agonist was administered subcutaneously, and the injection could be repeated if necessary. The selection of this time window (2nd to 5th days of the menstrual cycle) was based on the aim of initiating pituitary suppression early in the cycle, which is crucial for regulating ovarian function. By suppressing the pituitary gland’s secretion of gonadotropins during this period, we could better manipulate the ovarian response, including follicular development and hormone levels, thereby optimizing the conditions for IVF/ICSI treatment. The number of injections (≥ 1) was determined based on individual patient characteristics and the desired level of pituitary suppression, and was adjusted under careful monitoring. The long-acting GnRH agonist was injected for the second time if the anteroposterior uterine diameter was ≥ 70 mm after 30 days of injection. Once endometrium thickness ≤ 5 mm, serum estradiol ≤ 50 pg/mL, and luteinizing hormone (LH) ≤ 5 IU/L were observed after 30 days of the last long-acting GnRH agonist injection, controlled ovarian hyperstimulation was performed using gonadotropin (150–300 IU/day).

### GnRH antagonist protocol

In addition, women with infertility underwent the GnRH antagonist protocol with gonadotropin (150–300 IU/day) initiated 2–4 days after the menstrual cycle, with dosage adjustments based on age, BMI, and ovarian reserve. After this, a GnRH antagonist (0.25 mg/day) was administered if ≥ 1 follicle’s mean diameter was greater than 12–14 mm until the trigger day [11]. After the administration of the GnRH antagonist as described previously, ovulation was triggered under specific conditions. Follicular growth was monitored by transvaginal ultrasound, and ovulation was initiated when the leading follicles had grown to a diameter of 18–20 mm. Hormonal parameters, including LH, estrogen, and progesterone levels, were also monitored, and the ovulation was induced by an intramuscular injection of human chorionic gonadotropinat a dose of 5000–10,000 IU when both the follicular size and hormonal levels indicated optimal conditions for ovulation. This ensured the maturity of oocytes for retrieval and subsequent IVF/ICSI procedures.

### Endometrial preparation of frozen embryo transfer

Endometrial preparation protocols included natural, ovulation-promoting, and artificial cycles. The natural and ovulation-promoting cycles require continuous monitoring of the patient’s follicle development; thus, the cycle is more likely to be canceled due to the uncertainty of follicle development and excretion. Artificial cycle administration is convenient, and the cycle cancellation rate is low; thus, approximately 80% of patients use this regimen for endometrial preparation [17]. Estradiol valerate (4 mg/day) was administered orally from the second day of menstruation, and regular vaginal ultrasonography was performed to monitor endometrial growth and adjust medication. When the endometrial thickness reached 8 mm, the progesterone-transformed endometrium was administered, and the embryos were transplanted at a selected time. One or two embryos were transplanted according to the patient’s age, embryo quality, uterine dysplasia, cicatric uterus, and personal preference.

### ICSI protocol

The ICSI protocol involves several key steps. First, sperm preparation is carried out, which includes collecting sperm samples through appropriate methods. The collected sperm are then washed to remove seminal plasma using specialized solutions, and subsequently, they undergo capacitation in a carefully controlled environment, often involving incubation in specific media under precise temperature and chemical conditions to enhance their fertilizing ability. Next, oocyte retrieval is performed, typically using transvaginal ultrasound-guided aspiration, where a needle is guided by ultrasound to collect mature oocytes from the ovaries. Once the oocytes and capacitated sperm are ready, the micromanipulation process begins. The sperm are immobilized using micromanipulators under microscopic visualization to ensure stability and precision. Then, the immobilized sperm is carefully injected into the cytoplasm of the oocytes through a fine needle, which requires high levels of skill and precision to avoid damaging the oocytes. After successful injection, the injected oocytes, now considered embryos at an early stage, are cultured in a specialized embryo culture medium under optimal conditions, including a precisely controlled temperature, gas atmosphere, and nutrient supply, to support their development until they reach a stage suitable for further assessment, such as embryo transfer or cryopreservation. Throughout the ICSI protocol, strict quality control measures and adherence to medical ethics and regulatory requirements are essential to ensure the safety and success of the procedure, as it is a complex and delicate technique used in ART to assist couples with severe male factor infertility or other fertility issues.

### Outcome definition

The outcomes included biochemical pregnancy, clinical pregnancy, abnormal pregnancy, miscarriage, and live birth rate. Biochemical pregnancy was defined as serum β-HCG > 25 U/L 14 days after embryo transfer. Clinical pregnancy was defined as the presence of an intrauterine gestational sac, yolk sac, and original embryonic cardiac tube beat on ultrasonography 28–35 days after fresh embryo transfer. The miscarriage rate was defined as the termination of pregnancy with various non-human factors before 12 weeks of gestation (early spontaneous abortion) and the termination of pregnancy with non-human factors between 12 and 28 weeks of gestation (late abortion). The live birth rate was defined as the delivery of a living baby ≥ 28 weeks of pregnancy during the first embryo transfer, and it was calculated as the number of live birth cycles divided by the number of embryo transfer cycles.

### Statistical analysis

All the collected variables were classified as categorical or continuous. Categorical data were expressed as frequencies (percentages), and the differences between the GnRH antagonist and long-acting GnRH agonist protocols were compared using the *χ*^2^ test. Continuous data were presented as means (standard deviations) or medians (interquartile ranges) according to data distribution, and differences between groups were compared using the independent t-test or Kruskal–Wallis test. Both univariate and multivariate logistic regression analyses were applied to compare pregnancy outcomes between the GnRH antagonist and long-acting GnRH agonist protocols, presenting results as odds ratios (OR) with 95% confidence intervals (CI). All reported *p* values were 2-sided, and *p* values < 0.05 were considered statistically significant. Statistical analyses were performed using the SPSS software (release 21.0; SPSS Inc.).

## Results

### Clinical characteristics of patients

This study included 575 women with primary infertility: 501 women in the GnRH antagonist protocol group and 74 in the long-acting GnRH agonist protocol group. The overall ages of the women and men were 33.0 and 34.0 years, respectively. Among the study participants, 377 women underwent ICSI alone, 190 women underwent IVF alone, and 8 women underwent both ICSI and IVF. The baseline characteristics of the included patients are presented in Table 1. We noted significant differences between GnRH antagonist and long-acting GnRH agonist protocols for female age (*p* = 0.018), fertilization type (*p* = 0.007), therapeutic regimen (*p* < 0.001), BMI (*p* = 0.008), infertility duration (*p* = 0.042), basal follicle-stimulating hormone (FSH) (*p* = 0.002), and basal LH (*p* < 0.001). Moreover, no significant differences were observed in male age (*p* = 0.749), causes of infertility (*p* = 0.173), history of ovarian surgery (*p* = 1.000), basal E2 (*p* = 0.537), basal sinus follicles (*p* = 0.785), total sperm count (*p* = 0.501), sperm concentration (*p* = 0.229), and forward sperm rate (*p* = 0.272). Furthermore, the treatment and ovarian stimulation characteristics between the protocols were compared. There were no significant differences in total gonadotropin level (*p* = 0.117), total days of gonadotropin administration (*p* = 0.381), endometrial thickness on human chorionic gonadotropin (hCG) day (*p* = 0.881), mature ovum count (*p* = 0.121), number of transferred embryos (*p* = 0.077), and number of implanted embryos (*p* = 0.547). However, significant differences were observed in the total dose of FSH (*p* = 0.026), E2 on hCG day (*p* = 0.007), hCG follicle number (*p* = 0.020), and total ovum count (*p* = 0.019).

**Table 1.** The baseline characteristics of included patients.

Variables	Overall (*n* = 575)	Protocol		*p* Value
		GnRH antagonist (*n* = 501)	Long-acting GnRH agonist (*n* = 74)	
Female age (years)	33.00(30.00,36.00)	33.00(30.00,36.00)	31.50(30.00,35.00)	0.018
Male age (years)	34.00(31.00,39.00)	34.00(31.00,39.00)	35.00(32.00,38.75)	0.749
Fertilization type (%)				0.007
ICSI	377(65.57)	340(67.86)	37(50.00)	
ICSI+IVF	8(1.39)	6(1.20)	2(2.70)	
IVF	190(33.04)	155(30.94)	35(47.30)	
Therapeutic regimen (%)				<0.001
Falling FET	51(8.87)	47(9.38)	4(5.41)	
Antagonist protocol	62(10.78)	62(12.38)	0(0.00)	
Artificial cycle FET	457(79.48)	391(78.04)	66(89.19)	
Long-acting protocol	4(0.70)	0(0.00)	4(5.41)	
Natural cycle FET	1(0.17)	1(0.20)	0(0.00)	
BMI (kg/m^2^)	23.40(21.10,26.37)	23.50(21.20,26.70)	23.30(20.60,24.30)	0.008
Infertility duration (years)	3.00(1.38,5.00)	3.00(1.75,5.00)	2.00(1.00,4.00)	0.042
Causes of infertile (%)				0.173
Husband	66(11.48)	54(10.78)	12(16.22)	
Wife	342(59.48)	295(58.88)	47(63.51)	
Both	110(19.13)	102(20.36)	8(10.81)	
Unclear	57(9.91)	50(9.98)	7(9.46)	
History of ovarian operation (%)				1.000
No	548(95.30)	477(95.21)	71(95.95)	
Yes	27(4.70)	24(4.79)	3(4.05)	
Basal FSH	6.06(4.93,7.17)	6.15(5.07,7.26)	5.54(4.29,6.42)	0.002
Basal LH	4.40(2.96,6.42)	4.52(3.15,6.56)	2.75(1.34,5.09)	<0.001
Basal E2	37.42(26.91,48.56)	37.42(28.44,48.54)	37.53(22.82,48.67)	0.537
Basal sinus follicles	15.00(10.00,21.00)	15.00(9.00,21.00)	15.00(11.00,20.00)	0.785
Total sperm count	152.46(66.81,258.26)	151.24(70.36,251.63)	168.75(53.73,348.95)	0.501
Sperm concentration	44.07(20.25,81.50)	43.94(20.72,80.50)	58.98(19.43,100.83)	0.229
Forward sperm rate	47.00(30.00,60.00)	48.00(31.00,60.00)	46.00(21.00,61.00)	0.272
Total gonadotropin	2400.00(2100.00,2700.00)	2400.00(2100.00,2700.00)	2400.00(2062.50,2700.00)	0.117
Total days of gonadotropin	9.00(8.00,10.00)	9.00(8.00,10.00)	9.00(8.25,10.00)	0.381
Total dose of FSH	2400.00(2025.00,2700.00)	2400.00(2100.00,2700.00)	2325.00(2025.00,2625.00)	0.026
E2 on hCG day (pg/ml)	3000.00(1739.00,3000.00)	3000.00(1647.50,3000.00)	3000.00(2640.00,3000.00)	0.007
HCG follicle number	14.00(9.00,23.00)	14.00(8.00,22.00)	19.00(11.00,23.00)	0.020
Endometrial thickness on hCG day (mm)	1.10(0.90,1.20)	1.10(0.90,1.20)	1.10(0.91,1.20)	0.881
Total ovum count	13.00(8.00,21.00)	13.00(7.00,20.00)	18.00(10.00,21.00)	0.019
Mature ovum count	6.00(0.00,13.00)	6.00(0.00,13.00)	4.00(0.00,11.75)	0.121
Number of transferred embryos	1.00(1.00,2.00)	1.00(1.00,2.00)	2.00(1.00,2.00)	0.077
Number of implanted embryos	0.00(0.00,1.00)	0.00(0.00,1.00)	0.00(0.00,1.00)	0.547

### Pregnancy outcomes

The pregnancy outcomes in the GnRH antagonist and long-acting GnRH agonist protocol groups are presented in Table 2. The live birth rate was significantly higher in the long-acting GnRH agonist protocol group than in the GnRH antagonist protocol group (*p* = 0.009). However, no significant differences were observed between the two protocols for the biochemical pregnancy rate (*p* = 0.068), clinical pregnancy rate (*p* = 0.424), abnormal pregnancy rate (*p* = 1.000), and miscarriage rate (*p* = 0.495). Univariate logistic regression indicated that the GnRH antagonist protocol was associated with a lower live birth rate than the long-acting GnRH agonist protocol (OR: 0.501; 95%CI: 0.305–0.824; *p* = 0.009), whereas no significant differences were observed between the two protocols for biochemical pregnancy rate (OR: 6.331; 95%CI: 0.858–46.740), clinical pregnancy rate (OR: 0.794; 95%CI: 0.487–1.296), abnormal pregnancy rate (OR: 0.967; 95%CI: 0.420–2.230), and miscarriage rate (OR: 2.878; 95%CI: 0.379–21.820). After adjusting potential confounding factors, no significant differences were observed between the two protocols for biochemical pregnancy rate (OR: 6.331; 95%CI: 0.858–46.740), clinical pregnancy rate (OR: 1.223; 95%CI: 0.629–2.379), abnormal pregnancy rate (OR: 0.900; 95%CI: 0.290–2.795), miscarriage rate (OR: 2.878; 95%CI: 0.379–21.820), and live birth rate (OR: 0.757; 95%CI: 0.392–1.460) (Table 3).

**Table 2.** Pregnancy outcomes of two treatment protocols.

Outcomes	Overall (*n* = 575)	Protocol		*P* value
		GnRH antagonist (*n* = 501)	Long-acting GnRH agonist (*n* = 74)	
Biochemical pregnancy rate (%)				0.068
No	534(92.87)	461(92.02)	73(98.65)	
Yes	41(7.13)	40(7.98)	1(1.35)	
Clinical pregnancy rate (%)				0.424
No	293(50.96)	259(51.70)	34(45.95)	
Yes	282(49.04)	242(48.30)	40(54.05)	
Abnormal pregnancy rate (%)				1.000
No	522(90.78)	455(90.82)	67(90.54)	
Yes	53(9.22)	46(9.18)	7(9.46)	
Miscarriage rate (%)				0.495
No	555(96.52)	482(96.21)	73(98.65)	
Yes	20(3.48)	19(3.79)	1(1.35)	
Live birth rate (%)				0.009
No	398(69.22)	357(71.26)	41(55.41)	
Yes	177(30.78)	144(28.74)	33(44.59)	

**Table 3.** Univariate and multivariate logistic regression for pregnancy outcomes.

Outcomes	Crude OR (95% CI)	Adjusted OR (95% CI)
Biochemical pregnancy rate	6.331(0.858–46.740)	6.331(0.858–46.740)^a^
Clinical pregnancy rate	0.794(0.487–1.296)	1.223(0.629–2.379)^b^
Abnormal pregnancy rate	0.967(0.420–2.230)	0.900(0.290–2.795)^c^
Miscarriage rate	2.878(0.379–21.820)	2.878(0.379–21.820)^a^
Live birth rate	0.501(0.305–0.824)	0.757(0.392–1.460)^d^

^a^ Not variable entered in multivariate analysis.

^b^ Adjusted for female age, therapeutic regimen, and endometrial thickness on hCG day.

^c^ Adjusted for BMI and sperm concentration.

^d^ Adjusted for female age, and therapeutic regimen.

## Discussion

### Principal findings

Optimal controlled ovarian stimulation protocols are important to improve pregnancy and live birth rates and reduce adverse pregnancy outcomes for women with infertility undergoing IVF/ICSI. This study aimed to compare pregnancy outcomes between the GnRH antagonist and long-acting GnRH agonist protocols for women with infertility undergoing IVF/ICSI. This retrospective cohort study recruited 575 women with primary infertility undergoing IVF/ICSI who were treated using a GnRH antagonist protocol and a long-acting GnRH agonist protocol. The results showed that the long-acting GnRH agonist protocol was associated with an elevated total ovarian count. Moreover, the crude analysis found that the long-acting GnRH agonist protocol was associated with an increased live birth rate compared with the GnRH antagonist protocol. However, no significant differences were observed between the protocols in terms of biochemical pregnancy, clinical pregnancy, abnormal pregnancy, miscarriage, and live birth rates after adjusting for potential confounding factors.

### Results in the context of what is known

Several studies have compared pregnancy outcomes between the GnRH antagonist and long-acting GnRH agonist protocols for women with infertility undergoing IVF/ICSI. Geng et al. identified 1,883 consecutive IVF/ICSI fresh cycles in normal ovarian responders. They found that a long-acting GnRH agonist protocol was associated with elevated oocytes obtained, implantation or pregnancy rate, and lower LH level or E2/oocyte ratio on the day of hCG administration [10]. Liu et al. identified 95 patients undergoing IVF-embryo transfer (IVF-ET) and found that long-acting GnRH agonist protocols were associated with more oocyte retrieval, available embryos, and good-quality embryos [18]. Zhang et al. collected data from 282 women with adenomyosis undergoing their first IVF/ICSI and suggested a GnRH antagonist protocol and a long-acting GnRH agonist protocol with similar clinical pregnancy, live birth, and cumulative live birth rates for women with infertility [19]. Chen et al. retrospectively collected data from 8,579 women undergoing their first IVF-ET. They found that the long-acting GnRH agonist protocol was associated with an increased live birth rate compared to the GnRH antagonist protocol. The suitability of controlled ovarian stimulation protocols is dependent on the biological characteristics of women with infertility [20]. Ge et al. found an ultra-long or long protocol may be beneficial in infertile women with adenomyosis and received fresh embryo transfer [13]. Wu et al. suggested frozen embryo transfer following pretreatment with long-acting GnRH agonist has a better IVF/ICSI outcome and has potential advantages in terms of a lower gonadotropin dose and a shorter stimulation duration [9]. Xu et al. indicated depot GnRH agonist protocol improves the live birth rate per fresh embryo transfer cycle, but does not improve the cumulative live birth rate for infertile women with normal ovarian response [12]. Given the conflicting results of prior studies, including those mentioned above, and the recent discussions on the outcome of these protocols, we performed this study to compare pregnancy outcomes between the GnRH antagonist and long-acting GnRH agonist protocols for women with primary infertility undergoing IVF/ICSI. Our crude analysis suggested that the long-acting GnRH agonist protocol was associated with a higher total ovarian count and an increased live birth rate. However, as mentioned earlier, after adjusting for potential confounding factors, these differences were not statistically significant, indicating the need for caution in interpreting our results.

### Clinical implications

Our study found that the long-acting GnRH agonist protocol was associated with an increased live birth rate compared to the GnRH antagonist protocol. However, this result was non-significant after adjusting for potential confounding factors. Several reasons could explain this result [1]: The long-acting GnRH agonist protocol was superior to the GnRH antagonist protocol through the elimination of the fluctuation in preovulatory LH level during the ovarian hyperstimulation course [2,21]; The GnRH antagonist protocol was associated with an increased LH instability rate, which is negatively related to pregnancy rate [3,22–24]; Most women with infertility included in the study received GnRH antagonist protocol because it is associated with less complexity, mild ovarian stimulation, and a lower risk of ovarian hyperstimulation syndrome [22]; and [4] the prolonged administration of GnRH agonists for 3–6 months may be beneficial for patients with stage III/IV endometriosis, while highlighting that this intervention should not be performed in patients with mild to moderate endometriosis [25]. In addition, no significant differences were observed between the two protocols for biochemical pregnancy rate, clinical pregnancy rate, abnormal pregnancy rate, and miscarriage rate, which could be explained by the smaller number of expected outcomes, and the power was not enough to detect potential significant differences between protocols.

This study found that the long-acting GnRH agonist protocol was associated with a greater total ovum count. In contrast, no significant differences were observed between the two protocols for mature ovum count, number of transferred embryos, and number of implanted embryos. The potential reasons for this could be [1]: The steroid hormone pathway could affect the embryo environment and is associated with reduced oocytes and viable embryos [26]; and [2] The GnRH antagonist protocol was associated with lower LH levels, especially in the mid-follicular phase, and lower LH was negatively related to oocyte development in the early stage [10].

### Research implications

The results showed that the long-acting GnRH agonist protocol may improve live birth rate outcomes better compared to the GnRH antagonist and long-acting GnRH agonist protocols. This indicates that for women with infertility who want to achieve pregnancy through IVF/ICSI treatment, choosing a long-acting GnRH agonist protocol may lead to better pregnancy outcomes. This finding has an important guiding significance for clinicians in formulating treatment plans and providing patient counseling, which can help doctors optimize treatment strategies and improve pregnancy success rates and fertility opportunities for women with infertility.

### Strengths and limitations

The strengths of this study lie in the use of both univariate and adjusted analyses, which strengthen the validity and reliability of the findings. Adjustment for potential confounding factors ensured that the observed differences in pregnancy outcomes could be attributed to the protocol rather than other factors. In addition, this study has several shortcomings. First, because of its retrospective design, uncontrolled biases affecting the reliability of the results may be present. Second, female patients were older in the GnRH antagonist protocol group, which could have affected pregnancy outcomes. Third, the choice of controlled ovarian stimulation protocol was dependent on the ovarian reserve and age, whereas BMI was significantly related to an impaired ovarian response to exogenous gonadotropins [27,28]. Fourth, the background therapies in couples for improving pregnancy outcomes were not addressed, introducing potential bias in the therapeutic effects between GnRH antagonist protocol and long-acting GnRH agonist protocol for women with infertility undergoing IVF/ICSI. Finally, due to the relatively small sample size of patients who received the long-acting gonadotropin-releasing hormone agonist, this study did not perform subgroup analyses on patients based on cycle types (fresh or frozen-thawed).

## Conclusions

This study found that the long-acting GnRH agonist protocol might be superior to the GnRH antagonist protocol for increasing live birth rates in women with primary infertility undergoing IVF/ICSI. In contrast, differences in other pregnancy outcomes between both protocols, including biochemical pregnancy, clinical pregnancy, abnormal pregnancy, and miscarriage rates, were not statistically significant. Further large-scale prospective studies should be performed to compare pregnancy outcomes between the GnRH antagonist and long-acting GnRH agonist protocols for women with primary infertility undergoing IVF/ICSI.

## Data Availability

The datasets used and/or analysed during the current study are available from the corresponding author on reasonable request.
